# Fermented Alfalfa Meal Instead of “Grain-Type” Feedstuffs in the Diet Improves Intestinal Health Related Indexes in Weaned Pigs

**DOI:** 10.3389/fmicb.2021.797875

**Published:** 2021-12-13

**Authors:** Yuheng Luo, Yang Liu, Yuqing Shen, Jun He, Hua Li, Cong Lan, Jiayan Li, Hong Chen, Daiwen Chen, Zhihua Ren, Bing Yu, Zhiqing Huang, Ping Zheng, Xiangbing Mao, Jie Yu, Junqiu Luo, Hui Yan

**Affiliations:** ^1^Key Laboratory for Animal Disease-Resistance Nutrition of China Ministry of Education, Key Laboratory of Animal Disease-Resistant Nutrition and Feed of China Ministry of Agriculture and Rural Affairs, Key Laboratory of Animal Disease-Resistant Nutrition of Sichuan Province, Institute of Animal Nutrition, Sichuan Agricultural University, Chengdu, China; ^2^College of Food Science, Sichuan Agricultural University, Ya’an, China; ^3^Sichuan Province Key Laboratory of Animal Disease and Human Health, Key Laboratory of Environmental Hazard and Human Health of Sichuan Province, College of Veterinary Medicine, Sichuan Agricultural University, Chengdu, China

**Keywords:** weaned pigs, fermented alfalfa meal, intestinal barrier, colonic microbiota, immunity

## Abstract

Corn and soybean meal are the two main components in formula feed of farm animals, leading to a serious food competition between humans and livestock. An alternative may be to encourage the utilization of unconventional feedstuff in animal diet. In the current study, we evaluated the utilization of fermented alfalfa meal (FAM) in weaned pigs. Twenty weaned piglets (separately caged) were randomly divided into two groups. Pigs in the control group (CON) were fed corn-soybean meal diet, and part of corn and soya protein concentrate in the diet of another group was replaced by 8% FAM. After 40 days of feeding, the average feed intake of FAM pigs was increased (*P* > 0.05), and the villus height (VH) of jejunum and duodenum, crypt depth (CD), and VH/CD in FAM pigs was improved compared to the CON group (*P* < 0.05). The increase (*P* < 0.05) of goblet cells in the jejunum of FAM pigs was positively correlated with the expression of *MUC-2* gene (*R* = 0.9150). The expression of genes related to immunity (*IRAK4*, *NF*-κ*B*, and *IL-10*) and intestinal barrier (*Occludin* and *MUC-2*) in the jejunum, as well as the expression of *ZO-1* and *MUC-2* in the colon of these pigs, also showed increase (*P* < 0.05) compared to CON pigs, which was accompanied by the decrease (*P* < 0.05) of LPS concentration in the serum. The elevated proportion of CD3^+^ and CD8^+^ T-lymphocyte subsets in spleen (*P* < 0.05) confirmed the improvement of systemic immune function in FAM pigs. In addition, FAM pigs have a higher β-diversity of microbial community (*P* < 0.05) and promoted enrichment of probiotics such as *Lactobacillus* that positively was correlated with acetate concentration in the colon over CON pigs. In summary, partially replacement of expanded corn and soya protein concentrate with FAM (8%) may benefit the intestinal barrier and immune function of weaned pigs without affecting their growth. Our findings also provide evidence of the feasibility of FAM as a dietary component in pigs to reduce the consumption of grain.

## Introduction

The expanding livestock industry has led to an increase in the demand for grain and its by-products, resulting in deteriorating food competition between humans and livestock (van Kernebeek et al., 2016; van Zanten et al., 2018) and adverse environmental impacts (Schader et al., 2015). In recent years, to meet the increasing demand for high-quality meat, the swine populations in China have a rapid growth (Zhuang et al., 2020), leading to an increase in the consumption of corn-soybean meal based commercial feed. Replacing “grain-type” feedstuffs with “non-grain-type” feed resources is an effective way to ensure the sustainable development of the swine industry.

Alfalfa, especially *Medicago sativa* L., is regarded as “the queen of forages” because of its high content of protein, minerals, and vitamins and is the most common perennial legume forage in China (Shi et al., 2017). The annual global output of alfalfa is more than 300 million tons (Zheng et al., 2018). Alfalfa meal is commonly used in ruminants as a commercial feedstuff. However, due to its high content of crude fiber, alfalfa meal is not considered to be effectively used by pigs. Insoluble dietary fiber (IDF), such as cellulose, lignin, and xylan, is the main component of crude fiber in alfalfa, accounting for more than 90% of the total dietary fiber (Knudsen, 1997; Chen et al., 2013). For pigs, especially weaned piglets with limited tolerance to crude fiber, high levels of crude fiber from plant cell walls can reduce the digestibility of nutrients and the palatability of feed. We have previously reported that the solid-state fermentation by compound fungal strains can reduce the level of crude fiber in wheat bran and increase the content of soluble dietary fiber (SDF), and the supplement of fermented wheat bran in the diet can improve the intestinal health without affecting the growth performance in weaned pigs (Luo et al., 2021). The main purpose of the current study is to evaluate the feasibility of FAM replacing part of “grain-type” feedstuff in weaned pigs. Meanwhile, the growth performance, intestinal barrier, immunity related parameters, and the colonic microbiota were investigated to discuss the potential effects of FAM on intestinal health of weaned pigs.

## Materials and Methods

All experimental procedures and animal care were accomplished in accordance with the Guide for the Care and Use of Laboratory Animals provided by the Institutional Animal Care Advisory Committee for Sichuan Agricultural University. The experimental protocols used in the present study were approved by the Sichuan Agricultural University Institutional Animal Care and Use Committee No. 69130079.

### Animals and Diets

A total of 20 Duroc × Landrace × Yorkshire cross-bred weaned pigs with similar initial body weight (7.20 ± 0.27 kg) were randomly allocated to two groups with 10 replicates per group and 1 pig per replicate, and each pig was raised in a 1.5 m × 0.7 m × 1.0 m metabolism cage. Pigs in the control group (CON) were fed a basal corn-soybean meal diet. For pigs in another group (FAM), a total of 7.2% expanded corn and 1.8% soya protein concentrate in the diet were replaced by equivalent FAM, respectively. FAM used in the current study was prepared according to the method established in our previous study (Luo et al., 2021). The diets were formulated to meet the nutrient recommendations of the National Research Council (2012) and no antibiotics were supplemented (Table 1). The whole experiment lasted for 40 days, and the diet and water were available *ad libitum*. The feed intake, mental status, and health condition of each pig were investigated and recorded every day.

**TABLE 1:** The composition and the level of nutrients in the experimental diets (air-dried basis).

Item	CON	FAM
Ingredient (%)		
Expanded corn	37.19	29.99
Peeled soybean meal	20.55	20.55
corn starch	18.00	18.00
Fish meal	4.50	4.50
Soya protein concentrate	4.30	2.50
Whey powder	5.50	5.50
Sucrose	4.00	4.00
Glucose	3.00	4.00
Soybean oil	0.20	0.20
FAM	0.00	8.00
NaCl	0.30	0.30
*L*-Lysine⋅HCl	0.40	0.40
DL-Methionine	0.15	0.15
Threonine	0.14	0.14
Tryptophan	0.03	0.03
Limestone	0.60	0.60
Dicalcium phosphate	0.80	0.80
Chloride choline	0.10	0.10
Vitamin premix^1^	0.04	0.04
Mineral premix^2^	0.20	0.20
Total	100.00	100.00
Nutritional value^3^		
Digestible energy (DE, Mcal/kg)	3.53	3.55
Crude protein (CP, %)	17.45	16.66
Calcium (Ca, %)	1.00	0.79
Total phosphorus (TP, %)	0.63	0.56
Available phosphorus (AP, %)	0.43	0.40
Digestible lysine (D-Lys, %)	1.44	1.33
Digestible methionine (D-Met, %)	0.45	0.43
Methionine + Cystine (D-Met + D-Cys, %)	0.66	0.67
Digestible threonine (D-Thr, %)	0.86	0.81
Digestible tryptophan (D-Trp, %)	0.28	0.20
Crude fiber (CF, %)	1.92	6.64
Neutral detergent fiber (NDF, %)	12.12	14.12
Acid detergent fiber (ADF, %)	10.61	12.17
Soluble dietary fiber (SDF, %)	2.10	3.96
Insoluble dietary fiber (IDF, %)	7.83	8.47

*^1^The vitamin premix provided Vitamin A 30,000,000 IU, Vitamin D3 10,000,000 IU, Vitamin E 80,000 IU, Vitamin K3 10,000 mg, Vitamin B1 10,000 mg, Vitamin B2 25,000 mg, Vitamin B6 12,000 mg, Vitamin B12 120 mg, D-pantothenic acid 50,000 mg, folic acid 5,000 mg, and biotin 500 mg per kg of diet.*

*^2^The mineral premix (7–25 kg) provided, 350 mg Fe (FeSO_4_⋅H_2_O), 41.67 mg Cu (CuSO_4_⋅5H_2_O), 292.78 mg Zn (ZnSO_4_⋅7H_2_O), 66.20 mg Mn (MnSO_4_⋅H_2_O), 8.31 mg I (KI), 30.61 mg Se (Na_2_SeO_3_), and 1209.55 mg CaCO_3_ per kg of diet.*

*^3^The level of CP, CF, NDF, ADF, SDF, and IDF was measured value, while the content of DE, Ca, TP, AP, D-Lys, D-Met, D-Met + D-Cys, D-Thr, and D-Trp was calculated value.*

*CON, control, FAM, fermented alfalfa meal.*

### Sample Collection, RNA Extraction, and Real-Time PCR

On the early morning of day 41, all pigs were weighed and 10 mL jugular blood was collected for flow cytometry and biochemical analysis. After that, pigs were sacrificed under anesthesia by lethal injection of 200 mg/kg sodium pentobarbital. The abdomen of each pig was opened immediately and approximately 2 cm of middle duodenum, jejunum, ileum, and colon was collected and fixed in 100 mL 10% formaldehyde solution for histology analysis, respectively, and the mucosa of each segment was scraped using a sterilized slide and stored in liquid nitrogen for RNA extraction. Approximate 5 g of colonic digesta was collected for the analysis of microbiota and concentration of short chain fatty acids (SCFAs), and 5 g of spleen was also collected and stored on ice for the determination of T-lymphocyte subsets.

The RNA of mucosal samples of jejunum and colon from each pig were extracted using Trizol (TAKARA, Japan). The mRNA levels of key genes of pattern recognition receptor related signaling pathway, such as *NOD1*, *NOD2*, *RIPK-2* (Wan et al., 2018a), *TLR4*, *MyD88*, *TRAF6* (Wan et al., 2019), *IRAK4* (Islam et al., 2012), and *NF*-κ*B* (Tian et al., 2016), and cytokines and antimicrobial peptides, such as *IL-1*β, *IL-6*, *IL-10* (Feng et al., 2015), and *pBD1* (Cheng et al., 2015), as well as intestinal barrier related genes, such as *ZO-1*, *Occludin* (Wan et al., 2020), and *MUC-2* (Wan et al., 2018b), were quantified by real-time PCR and the relative expression of each gene was calculated using the 2^–ΔΔCt^ method (Reid et al., 2011). The extraction of RNA and procedures of real-time PCR have been described before (Luo et al., 2021).

### Histological and Flow Cytometry Analysis

The villus height (VH), crypt depth (CD), and VH/CD ratio of each small intestine sample was measured. The methods of embedding, staining, and microscopic observation of paraffin sections have been described before (Luo et al., 2021). The concentrations of lipopolysaccharide (LPS) and D-lactate in each serum sample were assayed using a porcine ELISA kit purchased from Wuhan Meimian Biological Technology Co., Ltd. (Hubei, China). The proportion of CD3^+^, CD4^+^, and CD8^+^T lymphocyte subsets in each blood sample was detected following our reported method (Luo et al., 2021).

### DNA Extraction, 16S rRNA Amplicons Sequencing, and Analysis of Short Chain Fatty Acids

The genomic DNA in each colonic sample was extracted using a bead-beating method (Zoetendal et al., 1998). The subsequent establishment of 16S rRNA amplicons library and bioinformatics analysis was performed following our described methods (Luo et al., 2017), and all reads were deposited in the National Center for Biotechnology Information (NCBI) and can be accessed in the Short Read Archive (SRA) under accession number PRJNA763735. The determination of acetate, propionate, butyrate, valerate, isobutyrate, and isovalerate concentration in colonic digesta also referred to our published methods (Luo et al., 2015). In brief, approximate 1 g of each digesta sample was suspended in 2 mL distilled water and vortexed, and then centrifuged (12,000 g) at 4°C for 10 min. The supernatant (1 mL) was then mixed with 0.2 mL metaphosphoric acid, and 1-μL supernatant was analyzed using a gas chromatograph (Varian, GC CP3800).

### Statistical Analysis

An IBM SPSS Statistics 27 software was used to check normal distribution of the data. Then, differences in intestinal morphology related parameters, number of goblet cells, intestinal permeability indexes, proportion of T-lymphocyte subsets, and concentration of short-chain fatty acids (SCFAs) between CON and FAM groups were analyzed with independent sample t-test. Differences were considered statistically significant when *P* < 0.05. The correlation between the concentration of each SCFA and bacterial species was calculated using Pearson’s correlational analysis and the results were visualized using the vegan, ggcor, and dplyr packages of R 4.0.1. The Pearson’s correlation between the number of goblet cells and relative expression of *MUC-2* gene in jejunum was analyzed and visualized using a ggplot2 package of R 4.0.1, while the relative expression of genes in jejunum and colon of pigs in the two groups was clustered using a pheatmap package.

## Results

### Influence of Fermented Alfalfa Meal on the Growth Performance and the Morphology of Small Intestine in Weaned Pigs

**FIGURE 1:**
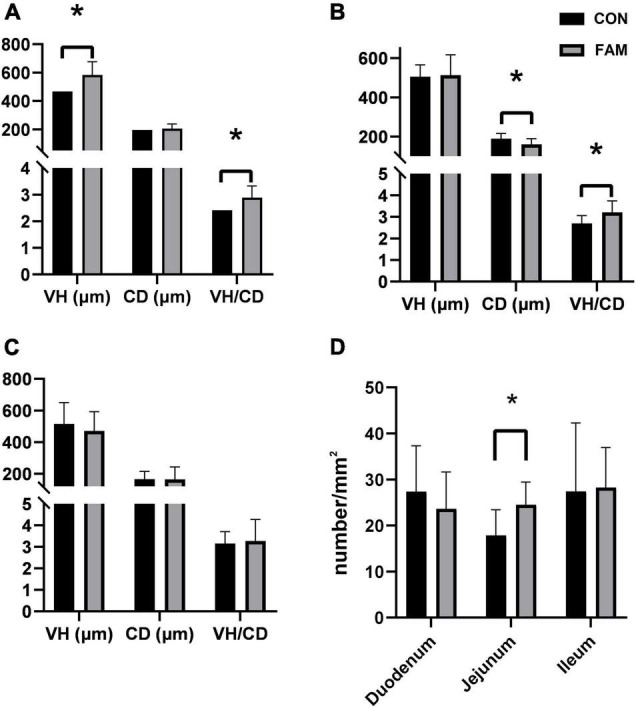
The morphology and number of goblet cells in the small intestine of the pigs. CON, control; FAM, fermented alfalfa meal. **(A–C)** The morphology of duodenum, jejunum, and ileum of the pigs. **(D)** The number of goblet cells per square millimeter of intestinal villus in each segment. *P < 0.05.

After 40 days of feeding, no difference in the final body weight (FBW), ADG, and F/G was found between the two groups (*P* < 0.05), but the ADFI of FAM pigs was increased (*P* < 0.05, Table 2). According to histological staining and scoring, comparing with CON pigs, the villus height (VH) in duodenum (Figure 1A), and the VH/CD in duodenum and jejunum (Figures 1A,B), except that in ileum (Figure 1C), was increased (*P* < 0.05). The number of goblet cells in jejunum (Figure 1D) was also increased (*P* < 0.05), while the crypt depth (CD) in the jejunum (Figure 1B) of FAM pigs was decreased (*P* < 0.05).

**TABLE 2:** The growth performance of the weaned pigs.

Item	CON	FAM	SD	*P*-value
IBW (kg)	7.23	7.17	0.27	0.22
FBW (kg)	19.22	17.21	2.13	0.08
ADG (g)	480.58	430.20	53.24	0.08
ADFI (g)	718.85	722.28	47.50	0.03
F/G	1.51	1.68	0.17	0.65

*IBW, initial body weight; FBW, final body weight; ADG, average daily gain; ADFI, average daily feed intake; F/G, feed/gain; CON, control; FAM, fermented alfalfa meal; SD, standard deviation.*

### Effect of Fermented Alfalfa Meal on the Intestinal Barrier and Immune Related Indexes in the Intestine of the Weaned Pigs

**FIGURE 2:**
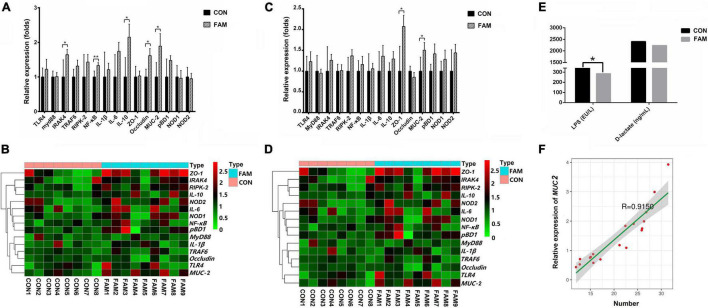
The relative expression of barrier and immunity relating genes and the permeability of intestine in the weaned pigs. **(A)** The relative expression of genes in jejunum. **(B)** The expression of genes in the jejunum of each pig visualized by heatmap. **(C)** The relative expression of genes in colon. **(D)** The expression of genes in the colon of each pig visualized by heatmap. **(E)** The concentration of LPS and D-lactate in serum. **(F)** The correlation between the relative expression of *MUC-2* and the number of goblet cells in jejunum. **P < 0.01 and *P < 0.05.

As shown above, the feeding of FAM containing diet affected the intestinal morphology of the weaned pigs, which may further influence the intestinal barrier and immune function of these animals. Because both the morphology and number of goblet cells in jejunum showed marked changes, we selected jejunum as the representative of small intestine. According to real-time PCR results, the relative expression of several intestinal barrier and immunity related genes were increased (*P* < 0.01 or 0.05) in the jejunum (Figures 2A,B) and colon (Figures 2C,D) of FAM pigs compared with CON pigs. Particularly, most of these up-regulated genes were found in the jejunum (*IRAK4*, *NF*-κ*B*, *IL-10*, *Occludin*, and *MUC-2*) and only two (*ZO-1* and *MUC-2*) presented in the colon. Consistent with this, the concentration of LPS in the serum of FAM pigs was lower (*P* < 0.05) than that in CON pigs (Figure 2E). Because goblet cells are the producer of mucin in the gut (Grondin et al., 2020), we further analyzed the correlation between the number of goblet cells and the relative expression of *MUC-2* in jejunum, indicating a significant (*R* = 0.92) correlation between them (Figure 2F).

### Effect of Fermented Alfalfa Meal on the Proportion of T-Lymphocyte Subsets in Serum and Spleen of the Weaned Pigs

**FIGURE 3:**
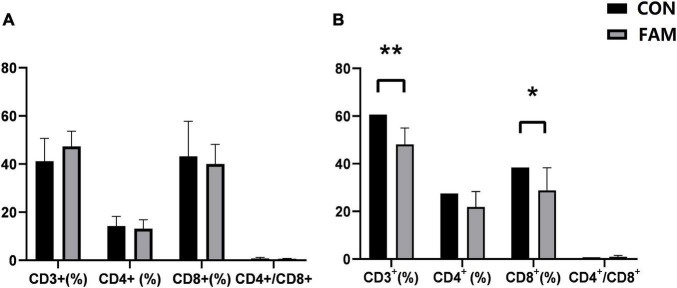
The percentage of different T lymphocyte subsets CD4^+^/CD8^+^ ratio in the blood and spleen of the weaned pigs. CON, control; FAM, fermented alfalfa meal. **(A)** Blood. **(B)** Spleen. **P < 0.01 and *P < 0.05, respectively.

The difference in the concentration of serum LPS may reflect the change in intestinal permeability and immunity of the pigs. We thus compared the proportion of T-lymphocyte subsets in the blood of pigs between the two groups. The results of flow cytometry showed that the proportion of CD3^+^ (*P* < 0.01) and CD8^+^ (*P* < 0.05) T lymphocyte subsets in the spleen of FAM pigs was decreased compared to CON pigs (Figure 3B). However, we did not observe any differences in the proportion of CD4^+^ T-lymphocyte subsets and ratio of CD4^+^/CD8^+^ in the blood (*P* > 0.05) of pigs between the two groups (Figure 3A).

### Effect of Fermented Alfalfa Meal on the Microbial Community and Metabolites in the Colon of the Weaned Pigs

**FIGURE 4:**
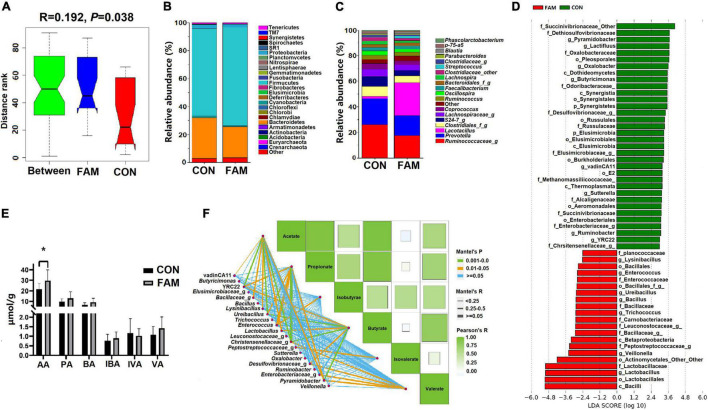
The microbial community, concentration of SCFAs, as well as the interaction between characteristic bacteria and SCFAs in the colon of the weaned pigs. **(A)** The β-diversity of colonic microbial community based on unweighted unifrac distance. **(B)** The composition of colonic microbiota at phylum level. **(C)** The relative abundance of top 20 microbial genera in the colonic digesta. **(D)** Bacterial groups with significant differences in relative abundance in the two groups of pigs, and data shows according to the results of Lefse analysis (characteristic bacteria). **(E)** The concentration of each SCFA in the colon of the pigs. **(F)** The correlation between the concentration of SCFAs and the relative abundance of characteristic bacteria. CON, control; FAM, fermented alfalfa meal. *P < 0.05.

The utilization of fiber in the hindgut of pigs mainly depends on the fermentation of microorganisms. Therefore, we examined the composition of microbial community and the concentration of main metabolites (SCFAs) in the colon of the pigs. Sequencing based on 16S rRNA amplicons showed no difference (*P* > 0.05) in the α-diversity of colonic microbial community between the two groups (Table 3), but a difference (*P* = 0.038) in β-diversity was found (Figure 4A).

**TABLE 3:** The α-diversity indexes of microbial community in the colonic digesta of the weaned pigs.

Index	CON	FAM	SD	*P*-value
Chao1	11825.18	10962.04	1898.304	0.55
Observed species	5088.77	5022.37	509.10	0.87
PD whole tree	310.58	309.38	28.27	0.96
Shannon	9.12	8.91	0.67	0.72

*CON, control; FAM, fermented alfalfa meal; SD, standard deviation.*

Except for those unidentified phyla, a total of 22 and 25 known phyla were identified in the colonic digesta of CON and FAM pigs (Figure 4B), respectively. Of these phyla, Firmicutes (62.63∼70.38%), Bacteroidetes (22.01∼29.10%), and Proteobacteria (2.13∼2.60%) were the most predominant bacteria in relative abundance. The difference of colonic microbial composition between the two groups was mainly reflected in the genus level. Among the top 20 genera, the abundance of *Lactobacillus* showed significantly different (*P* = 0.013) between the two groups, which was absolutely higher in FAM (22.58%) pigs compared to CON (1.48%) pigs (Figure 4C). Linear discriminant analysis (LDA) and LDA coupled with effect size (LefSe, α = 0.01, LDA score > 2.0) showed 34 and 21 taxa enriched in CON and FAM pigs (Figure 4D), respectively. Of these taxa, 8 known genera including *Pyramidobacter*, *Lactifluus*, *Oxalobacter*, *Buryricimonas*, *vadinCA11*, *Sutterella*, *Ruminobacter*, and *YRC22* were particularly abundant in CON pigs, while 7 other known genera, *Lysinibacillus*, *Enterococcus*, *Ureibacillus*, *Bacillus*, *Trichococcus*, *Veillonella*, and *Lactobacillus*, were specifically abundant in FAM pigs (Figure 4D).

Among the six measured SCFAs, the concentration of acetate showed increased (*P* < 0.05) in the colonic digesta of FAM pigs compared to CON pigs (Figure 4E). Further Pearson’s correlation showed multiple correlations between the abundance of specifically enriched taxa and the concentration of SCFAs (Figure 4F), highlighting the contribution of these bacteria to microbial metabolites in the colon of these pigs.

## Discussion

In the past, the application of forage grass was mostly limited to ruminants. The digestive physiological structure of monogastric livestock such as pig determines that they cannot effectively use forage. The rational development of non-grain feed resources is a feasible way to remit the contradiction between increasing population and the shortage of food. In the current study, we proved the feasibility of small-amount substitution of expanded corn and soybean protein concentrate by FAM in the feed of weaned piglets.

Limited studies focused on the utilization of alfalfa meal in swine feed. For example, the daily gain of growing pigs housed in a cold (10°C), thermoneutral (22.5°C), or hot (35°C) thermal environment can be reduced by 1, 3, and 5% by the supplement of 10% dehydrated alfalfa meal in their feed (Stahly and Cromwell, 1986). Similar result is also found in sows fed 20% alfalfa meal containing feed (Pond et al., 1980). When the proportion of alfalfa meal in feed ranges from 20 to 60%, its negative effect on daily gain and feed/gain in growing-finishing pigs is dose-dependent (Powley et al., 1981), which may be due to the high concentration of crude fiber in alfalfa meal. It is worth noting that the alfalfa meal used in these studies is not specially processed (e.g., fermentation). In this study, the proportion of FAM was 8%, resulting in an increase of crude fiber from 1.95 (CON) to 3.64 (FAM) in formula feed. Meanwhile, the concentration of extruded corn and soya protein concentrate in the feed of FAM group was 7.2 and 1.8% lower than that of CON group. However, no significant changes in most growth performance related parameters, except for increased ADFI, were found in FAM pigs after a continuous feeding for 40 days. These findings suggest that partially replacing expanded corn and soybean protein with 8% FAM has no effect on the growth of weaned pigs.

Our study highlighted the role of FAM containing diet on intestinal health of the weaned pigs. The intestinal epithelium has strong plasticity (de Sousa E Melo and de Sauvage, 2019). Changes in villus density and height, crypt depth, as well as the renewal rate of epithelium are regarded as common parameters to assess host response to nutrients, pathogens, and stress (Kopf and Sixt, 2019). Crypt in intestinal epithelium is considered as a villus workshop due to its internal stem cells (De Gregorio et al., 2018). We found that comparing with pigs fed basal diet, pigs fed FAM containing diet had higher villus height and ratio of villus height/crypt depth, especially in jejunum and duodenum, indicating a promotion of FAM on the development of villi in the small intestine. Interestingly, we further found that the number of goblet cells in the jejunum of pigs fed FAM diet is remarkably higher than that of pigs in the control group, which was positively correlated with the expression of *MUC-2* gene. It is well known that goblet cells, existing in the single columnar epithelium of small and large intestine, secrete mucin 2 (Pelaseyed et al., 2014) that works with water, inorganic salt, and antibacterial peptide to form viscous gel-like network in the gut (Kim and Ho, 2010). Our result thus suggests an enhanced intestinal mucus barrier in pigs fed FAM. In addition, the increased expression of tight junction protein (e.g., Occludin and ZO-1) genes in the jejunum and colon suggested an improved intestinal mechanical barrier in pigs fed FAM containing diet. The lower concentration of LPS in the serum provides further evidence for FAM diet improving the intestinal barrier function of these pigs.

As the “pioneer” of pathogens invading body, LPS triggers the immune response of the host by activating the signal pathway related to pattern recognition receptors such as TLR4 (Szabo et al., 2010). We found that after feeding FAM containing diet, the expression of *IRAK4*, *NF*-κ*B*, and *IL-10* genes in the jejunum of pigs was elevated compared to the CON group. IRAK4 can activate downstream inhibitor of NF-κB kinase (IKK) and mitogen activated protein kinase (MAPK) pathway by inducing the binding of IRAK1 and tumor necrosis factor-associated factor 6 (TRAF6) (Madera-Salcedo et al., 2013), which in turn promotes the nuclear translocation of NF-κB (Sina et al., 2010). Among all NF-κB-induced cytokines, IL-10 is a well-known anti-inflammatory cytokine that can stimulate natural and specific immunity (de Vries, 1995). Therefore, our findings indicate that the supplement of FAM in the diet may enhance the immune function of the weaned pigs through MyD88 dependent LPS/TLR4 signal pathway. This inference was also confirmed by the results of flow cytometry, that is, the proportion of CD3^+^ and CD8^+^ T lymphocytes in the spleen of pigs fed FAM diet was decreased compared to control.

Gut microbiota constitutes another important intestinal barrier. The composition of microflora is an important factor affecting intestinal homeostasis of the host (Ohland and Jobin, 2015). In the current study, 16S rRNA based amplicon sequencing revealed similar α-diversity but different composition of microbial community in the colon between FAM and CON pigs. Genera that may contain large amounts of probiotics, such as *Lactobacillus* and *Enterococcus*, were enriched in the colon of FAM pigs. Interestingly, *Lysinibacillus*, a genus with a strong ability to degrade lignocellulose (Ayeronfe et al., 2019), was also found more abundant in pigs fed FAM diet. It is speculated that the changed microbiota in colon of FAM pigs contributed to the fermentation ability more than CON pigs, which was confirmed by the markedly higher concentration of acetate in the colon of these pigs. A study in growing pigs also shows that with the increase of alfalfa meal in the diet, the pH value of ileal digesta and the concentration of acetate in feces is increased (Chen et al., 2013). Our results of correlation analysis proved that the distinct microbial species enriched in the colon of the pigs contributed to the different concentration and composition of SCFAs between the two groups. Moreover, although the level of crude fiber in FAM diet was as high as 6.64% (vs. 1.92% in CON diet), no decrease in the growth performance of the animals was observed in this study, which may be related to the improvement of microbial composition and metabolites in the hindgut. However, the direct effect of the dietary fiber composition of FAM on intestinal microbiota in pigs remains to be explored.

## Conclusion

In the current study, we showed that the supplement of FAM in the diet improved the morphology and reduced the mucosal permeability of small intestine without affecting the growth of weaned piglets. The interaction between increased number of goblet cells and *MUC-2* expression in jejunum suggests that FAM containing diet may promote the secretion of mucus in the piglets. We also found that the supplement of FAM may improve the internal environment of hindgut by shape the composition and metabolites of microbiota. Our findings provide powerful evidences that FAM can be used in the feed of weaned pigs, and the proportion of FAM in the formula diet can reach at least 8% by replacing equivalent expanded corn and/or soya protein concentrate.

## Data Availability Statement

The datasets presented in this study can be found in online repositories. The names of the repository/repositories and accession number(s) can be found below: https://www.ncbi.nlm.nih.gov/genbank/, PRJNA763735.

## Ethics Statement

The animal study was reviewed and approved by Institutional Animal Care Advisory Committee for Sichuan Agricultural University.

## Author Contributions

YuL designed the experiment, wrote the manuscript, and provided funds. YaL, YS, and JH finished the animal trial and laboratory analysis. CL, JiL, and HL finished the real-time PCR and bioinformatics analysis. HC, DC, ZR, and BY helped to design the animal trial and revise the manuscript. JH, ZH, PZ, XB, and JY helped to finish the laboratory analysis. JuL and HY helped to revise the manuscript. All authors contributed to the article and approved the submitted version.

## Conflict of Interest

The authors declare that the research was conducted in the absence of any commercial or financial relationships that could be construed as a potential conflict of interest.

## Publisher’s Note

All claims expressed in this article are solely those of the authors and do not necessarily represent those of their affiliated organizations, or those of the publisher, the editors and the reviewers. Any product that may be evaluated in this article, or claim that may be made by its manufacturer, is not guaranteed or endorsed by the publisher.
